# A nomogram model based on clinical markers for predicting malignancy of ovarian tumors

**DOI:** 10.3389/fendo.2022.963559

**Published:** 2022-11-24

**Authors:** Bingsi Gao, Xingping Zhao, Pan Gu, Dan Sun, Xinyi Liu, Waixing Li, Aiqian Zhang, Enuo Peng, Dabao Xu

**Affiliations:** ^1^ Department of Obstetrics and Gynecology, The Third Xiangya Hospital of Central South University, Changsha, Hunan, China; ^2^ Department of Obstetrics and Gynecology, The Obstetrics and Gynecology Hospital of Fudan University, Shanghai, China; ^3^ Department of Obstetrics and Gynecology, Hunan Provincial Maternal and Child Health Hospital, Changsha, Hunan, China

**Keywords:** ovarian tumors, malignant, ovarian cancer, clinical markers, prognostic nomogram model

## Abstract

**Objective:**

The aim of this study was to build a nomogram based on clinical markers for predicting the malignancy of ovarian tumors (OTs).

**Method:**

A total of 1,268 patients diagnosed with OTs that were surgically removed between October 2017 and May 2019 were enrolled. Clinical markers such as post-menopausal status, body mass index (BMI), serum human epididymis protein 4 (HE4) value, cancer antigen 125 (CA125) value, Risk of Ovarian Malignancy Algorithm (ROMA) index, course of disease, patient-generated subjective global assessment (PG-SGA) score, ascites, and locations and features of masses were recorded and analyzed (*p* 0.05). Significant variables were further selected using multivariate logistic regression analysis and were included in the decision curve analysis (DCA) used to assess the value of the nomogram model for predicting OT malignancy.

**Result:**

The significant variables included post-menopausal status, BMI, HE4 value, CA125 value, ROMA index, course of disease, PG-SGA score, ascites, and features and locations of masses (*p* 0.05). The ROMA index, BMI (≥ 26), unclear/blurred mass boundary (on magnetic resonance imaging [MRI]/computed tomography [CT]), mass detection (on MRI/CT), and mass size and features (on type B ultrasound [BUS]) were screened out for multivariate logistic regression analysis to assess the value of the nomogram model for predicting OT malignant risk (*p* 0.05). The DCA revealed that the net benefit of the nomogram’s calculation model was superior to that of the CA125 value, HE4 value, and ROMA index for predicting OT malignancy.

**Conclusion:**

We successfully tailored a nomogram model based on selected clinical markers which showed superior prognostic predictive accuracy compared with the use of the CA125, HE4, or ROMA index (that combines both HE and CA125 values) for predicting the malignancy of OT patients.

## Background

Ovarian tumors (OTs) are abnormal growths on the ovaries, which can be noncancerous (benign) or cancerous (malignant, ovarian cancer) (1). Among these, ovarian cancer (OC) is the eighth most common gynecological cancer and is considered the ‘silent killer’ of women worldwide due to its high mortality in the context of non-specific, early-stage symptoms (2, 3). The majority of OC patients are diagnosed at the advanced stages, and achieve poor 5-year survival outcomes despite comprehensive treatments (4). Therefore, timely therapeutic intervention, especially early diagnosis, is vital for the improvement of OC prognosis (5).

A variety of methods, including palpation, pelvic imaging modalities, and clinical serum biomarkers, such as cancer antigen 125 (CA125; also known as mucin 16 [MUC16]), human epididymis secretory protein 4 (HE4), and the Risk of Ovarian Malignancy Algorithm (ROMA) combining HE4 and CA125, have been reported for OT diagnosis (6). However, due to their limited sensitivity (SN) and specificity (SP), they have been unreliable in distinguishing malignant from benign OTs (7). Furthermore, menopausal state, mass features, ascites, and imaging methods might bias the diagnosis of malignancy (7–10). Although benign OTs are normally managed at local hospitals, OC is preferably treated at gynecological centers by oncological experts to achieve better outcomes (11). Therefore, an improved ability to differentiate between malignant and benign OTs would be of great importance for OT patients.

In this study, we screened out significant clinical variables associated with OT malignancy and built a nomogram model for predicting malignancy based on those markers.

## Materials and methods

This was a retrospective, diagnostic study conducted at the Gynecology Center of the Third Xiangya Hospital, which has oncological expertise. All participants were well-informed and signed written informed consent forms. This study was approved by the Ethics Committee of the Third Xiangya Hospital of Central South University (IRB No. 2018-S355). All methods were carried out following relevant guidelines and regulations.

A total of 1,268 patients diagnosed with an OT, either by type B ultrasound (BUS), computed tomography (CT), or magnetic resonance imaging (MRI) were enrolled between October 2017 and May 2019. Patients with non-ovarian, active cancer and a history of chemotherapy and/or radiotherapy who had experienced serious heart, liver, or kidney disease, or diabetes were excluded (12, 13). Based on the absence of menstrual periods for 12 months or an age older than 55 years, all patients were divided into post-menopausal or pre-menopausal groups. All tumors were preliminarily diagnosed through frozen pathology before being surgically removed, and post-operative specimen samples were evaluated by at least 2 gynecological pathologists. All participants had blood samples (5 mL/person) collected, processed, and further stored at -80°C until analysis. Serum HE4 and CA125 concentrations were measured on the cobas e411 analyzer (Elecsys; Roche Diagnostics, Mannhein, Germany) using the electrochemiluminescence technique according to strict standard protocols (14, 15). The detection ranges were 15.0–1500 pmol/L and 0.600–5000 U/mL for HE4 and CA125, respectively, as described in a previous study (16). The ROMA scores were calculated following the logistic regression analysis as described before (16):

premenopausal women, PI=−12.0+2.38×LN [HE4]+0.0626×LN [CA125];
postmenopausal women, PI=−8.09+1.04×LN [HE4]+0.732×LN [CA125];
and ROMA (%) = exp(PI)/[1+exp(PI)]×100.

### Statistics

Statistical analysis was performed with the Statistical Analysis System (SAS) v. 9.4 statistical software (SAS Institute Inc., Cary, NC, USA). Differences between the benign and the malignant groups were tested using a chi-squared test or Fisher’s exact test, as appropriate, and a *p-*value of 0.05 was considered significant. Multivariate logistic regression analysis was applied to decide which were the dominant variables for the establishment of the benign or malignant prediction models. The nomogram model for predicting OT malignancy was formulated with potential risk factors (*p* 0.05) based on the results of multivariate analysis, and its predictive performance was further measured by the decision curve analysis (DCA).

## Results

### Clinical characteristics and univariate logistic statistics of benign and malignant ovarian tumor patients

Of the 1,268 OT participants, 744 were premenopausal and 230 were postmenopausal patients with benign tumors, while 99 were premenopausal and 195 were postmenopausal patients with malignant tumors. The significant clinical variables included post-menopausal status, body mass index (BMI) level, HE4 value, CA125 value, ROMA index, course of disease, patient-generated subjective global assessment (PG-SGA) score, ascites, and mass locations and features (*p* 0.05). Women with OTs and mass mobility, a mass boundary, mass detection, and mass size were enrolled for univariate logic analysis.

In the comparison of women with benign tumors, the malignant group had higher post-menopausal status (*p* 0.0001), higher HE4 level (*p* 0.0001), higher CA125 value (*p* 0.0001), elevated ROMA index (*p* 0.0001), shorter course of disease (*p* 0.0001), more BMI ≥26 (*p* 0.0001), higher PG-SGA (*p =* 0.0002), and higher cachexia rate (*p* = 0.0002). Besides, there were significant difference regarding the ascites detected by palpation and BUS (all *p* 0.0001), solid and mixed mass detected by palpation and BUS (all *p* 0.0001), biliteral masses detected by BUS and MRI/CT (*p* = 0.0034 and *p* = 0.0152), larger mass size detected by BUS and MRI/CT (all *p* 0.0001), unclear/blurred mass boundary detected by BUS and MRI/CT (all *p* 0.0001). However, compared with screening by BUS, the detection rates of benign tumors and ascites were higher when screened using MRI/CT for mass and ascites detection, respectively (*p* 0.0001 and *p* = 0.0069) (**Table 1**).

**Table 1:** Characteristics and univariate statistics of benign and malignant ovarian tumor patients.

Variables	Classification	Benign	Malignant	*p*-value
Post-menopause	Yes (N, %)	230 (23.61%)	195 (66.33%)	0.0001
	No (N, %)	744 (76.39%)	99 (33.67%)	
	Total (N)	974	294	
HE4 (pmol/L)	Mean ± SD	64.2 ± 127.3	514.0 ± 899.1	0.0001
	Median (Min, Max)	52.9 (11.6, 3118.0)	138.7 (27.2, 7540.0)	
	Total (N)	808	244	
CA125 (U/mL)	Mean ± SD	24.7 ± 45.73	999.6 ± 1997.7	0.0001
	Median (Min, Max)	16.4 (0.81, 811.5)	203.9 (5.45, 12897.0)	
	Total (N)	819	254	
ROMA index	Mean ± SD	11.2 ± 9.8	59.4 ± 36.2	0.0001
	Median (Min, Max)	8.9 (1.0, 99.9)	69.0 (3.1, 100.0)	
	Total (N)	805	242	
Course of disease (days)	Mean ± SD	394.4 ± 856.8	147.7 ± 496.0	0.0001
	Median (Min, Max)	60 (1, 9125)	20 (1, 3650)	
	Total (N)	940	254	
BMI value	Mean ± SD	22.5 ± 3.0	22.5 ± 3.2	0.9113
	Median (Min, Max)	22.2(15.8, 33.8)	22.2(14.2, 30.9)	
	Total (N)	645	159	
BMI level	26 (N, %)	569 (88.2%)	137 (86.16%)	0.0001
	≥26 (N, %)	76 (11.78%)	32 (13.84%)	
	Total (N)	645	159	
PG-SGA	0-1 (N, %)	44 (4.68%)	6 (2.41%)	0.0002
	2-3 (N, %)	896 (95.32%)	239 (95.98%)	
	3 (N, %)	0 (0.0%)	4 (1.61%)	
	Total (N)	940	249	
Ascites (palpation)	Yes (N, %)	15 (1.6%)	45 (18.0%)	0.0001
	No (N, %)	925 (98.4%)	205 (82.0%)	
	Total (N)	940	250	
Mass locations (palpation)	Unilateral (N, %)	793 (94.52%)	184 (92.93%)	0.3893
	Bilateral (N, %)	46 (5.48%)	14 (7.07%)	
	Total (N)	839	198	
Mass features (palpation)	Cystic (N, %)	495 (79.3%)	29 (22.5%)	0.0001
	Solid (N, %)	68 (10.9%)	79 (61.2%)	
	Mixed (N, %)	61 (9.8%)	21 (16.3%)	
	Total (N)	624	129	
Mass mobility (palpation)	Good (N, %)	143 (31.5%)	30 (25.9%)	0.4993
	Moderate (N, %)	61 (13.4%)	17 (14.7%)	
	Poor (N)	250 (55.1%)	69 (59.5%)	
	Total (N)	454	116	
Mass locations (BUS)	Unilateral (N, %)	802 (91.1%)	171 (84.2%)	0.0034
	Bilateral (N, %)	78 (8.9%)	32 (15.8%)	
	Total (N)	880	203	
Mass size (BUS, diameter, mm)	Mean ± SD	71.8 ± 47.1	106.7 ± 51.2	0.0001
	Median (Min, Max)	62 (8,863)	104 (14,330)	
	Total (N)	885	201	
Mass features (BUS)	Cystic (N, %)	426 (51.3%)	48 (28.7%)	0.0001
	Solid (N, %)	46 (5.5%)	23 (13.8%)	
	Mixed (N, %)	358 (43.1%)	96 (57.5%)	
	Total (N)	830	167	
Mass boundary (BUS)	Clear (N, %)	546 (70.5%)	64 (39.8%)	0.0001
	Blurred (N, %)	109 (14.1%)	59 (36.7%)	
	Unclear (N, %)	120 (15.5%)	38 (23.6%)	
	Total (N)	775	161	
Mass detection (BUS)	Yes (N, %)	363 (48.7%)	66 (52.8%)	0.3915
	No (N, %)	383 (51.3%)	59 (47.2%)	
	Total (N)	746	125	
Ascites (BUS)	Yes (N, %)	74 (9.2%)	67 (36.6%)	0.0001
	No (N, %)	733 (90.8%)	116 (63.4%)	
	Total (N)	807	183	
Mass locations (MR/CT)	Unilateral (N, %)	493 (85.1%)	149 (77.6%)	0.0152
	Bilateral (N, %)	86 (14.9%)	43 (22.4%)	
	Total (N)	579	192	
Mass size (MR/CT)	Mean ± SD	83.2 ± 69.7	110.8 ± 84.4	0.0001
	Median (Min, Max)	67 (4,820)	94 (10,790)	
	Total (N)	582	193	
Mass boundary (MR/CT)	Clear (N, %)	398 (88.3%)	54 (38.6%)	0.0001
	Blurred (N, %)	22 (4.9%)	51 (36.4%)	
	Unclear (N, %)	31 (6.9%)	35 (25.0%)	
	Total (N)	451	140	
Mass detection (MR/CT)	Yes (N, %)	267 (52.0%)	25 (14.1%)	0.0001
	No (N, %)	247 (48.0%)	152 (85.9%)	
	Total (N)	514	177	
Ascites (MR/CT)	Yes (N, %)	98 (17.7%)	12 (8.5%)	0.0069
	No (N, %)	455 (82.3%)	130 (91.5%)	
	Total (N)	553	142	

The t or t’ test was used for quantitative data and the chi-square test was used for qualitative data.

P 0.05, significant difference; p 0.05, nonsignificant difference.

N, number; HE4, human epididymis protein 4; CA125, human carbohydrate antigen 125/mucin-16; ROMA, the Risk of Ovarian Malignancy Algorithm; SD, standard deviation; Min, minimum; Max, maximin; BMI, body mass index; BUS, ultrasound type B; MR, magnetic resonance; CT, computed tomography; PG-SGA, Patient-generated subjective global assessment.

### Multivariate logistic regression analysis for malignant ovarian tumors

On the basis that a malignant tumor was the dependent variable, a multivariate analysis was performed using the significant independent variables (*p* 0.05). The following factors, including the ROMA index, BMI ≥26, unclear/blurred mass boundary (MRI/CT), mass detection (MRI/CT), mass size (BUS), and mass features (BUS), were finally entered into the logistic model (**Tables 2A, B**). Risk factors for malignant OT (**Table 2C**) were: BMI ≥ 26 (OR [odds ratio] = 7.29, 95% confidence interval [CI]: 1.775 to 29.975), unclear mass boundary (OR = 3.07, 95% CI: 0.513 to 18.355), blurred mass boundary (OR = 9.20, 95% CI: 1.92 to 44.06), mass detectable by MRI/CT or BUS, respectively (OR = 4.23, 95% CI: 1.050 to 17.082 and OR = 3.26, 95% CI 0.823, 12.871), and solid masses (OR = 17.75, 95% CI: 1.901 to 165.655) or mixed masses (OR = 4.64, 95% CI: 1.323 to 16.296).

**Table 2A:** Multivariate logistic regression analysis. Type 3 effect analysis.

Effect	DF	Wald ChiSq	Pr > ChiSq
**ROMA index**	1	22.9318	<.0001
**BMI level**	1	7.5923	0.0059
**Mass Boundary (MR/CT)**	2	8.2467	0.0162
**Mass detection (MR/CT)**	1	4.1170	0.0425
**Mass size (BUS)**	1	7.9207	0.0049
**Mass features (BUS)**	2	8.6680	0.0131
**Mass detection (BUS)**	1	2.8300	0.0925

DF, degree of freedom; ChiSq, chi-square test; Pr, Pearson; BMI, body mass index; BUS, ultrasound type B; MR, magnetic resonance; CT, computed tomography.

**Table 2B:** Multivariate logistic regression analysis.

Varies	DF	Estimate	SE	WaldChiSq	Pr ChiSq
Intercept		-7.6388	1.2299	38.5782	.0001
ROMA index	1	0.0788	0.0165	22.9318	.0001
BMI level (26)	1	1.9870	0.7211	7.5923	0.0059
Mass Boundary (MR/CT)-unclear	1	1.1210	0.9127	1.5085	0.2194
Mass Boundary (MR/CT)-blurred	1	2.2193	0.7991	7.7124	0.0055
Mass detection (MR/CT)	1	1.4436	0.7115	4.1170	0.0425
Mass size (BUS)	1	0.00819	0.00291	7.9207	0.0049
Mass features (BUS)-solid	1	2.8761	1.1397	6.3680	0.0116
Mass features (BUS)-mixed	1	1.5344	0.6402	5.7437	0.0165
Mass detection (BUS)	1	1.1801	0.7015	2.8300	0.0925

SE, standard error; DF, degree of freedom; ChiSq, chi-square test; Pr: Pearson; BMI, body mass index; BUS, ultrasound type B; MR, magnetic resonance; CT, computed tomography.

Maximum likelihood estimation.

**Table 2C:** Multivariate logistic regression analysis.

Effect	Varies	Point estimate	Wald 95% CI	
ROMA index	/	1.082	1.048	1.117
BMI	≥26 vs <26	7.293	1.775	29.975
Mass Boundary (MR/CT)	unclear vs clear	3.068	0.513	18.355
Mass Boundary (MR/CT)	blurred vs clear	9.201	1.921	44.058
Mass detection (MR/CT)	yes vs no	4.236	1.050	17.082
Mass size (BUS)	/	1.008	1.002	1.014
Mass features (BUS)	solid vs cystic	17.745	1.901	165.655
Mass features (BUS)	mixed vs cystic	4.639	1.323	16.269
Mass detection (BUS)	yes vs no	3.255	0.823	12.871

BMI, body mass index; BUS, ultrasound type B; MR, magnetic resonance; CT, computed tomography.

Odds ratio estimation.

### The nomogram for predicting the malignant risk of OTs

**Figure 1:**
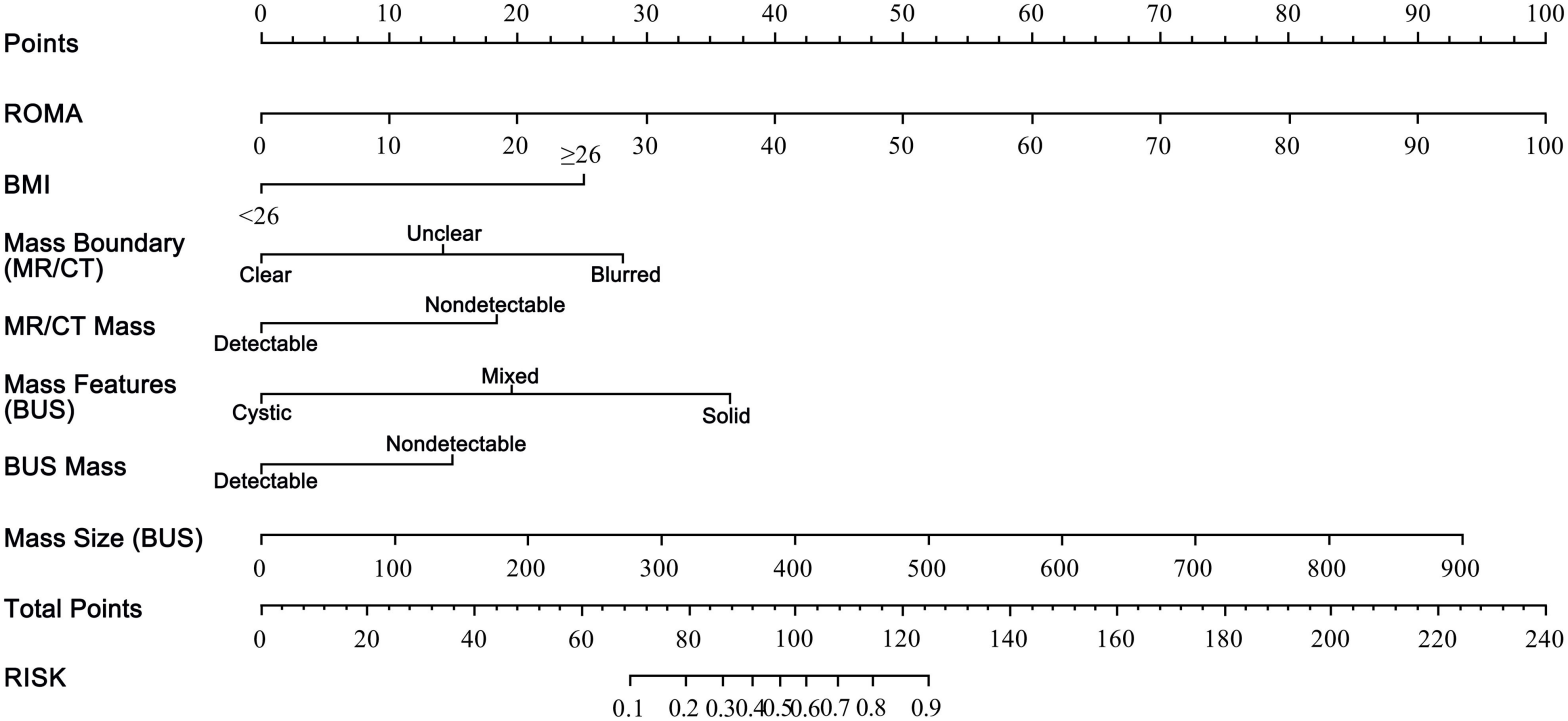
The nomogram for predicting malignant risk of ovarian tumors.The nomogram was developed in the primary cohort, with the variables of ROMA score, BMI ≥26, blurred and unclear boundary (MR/CT), mass detection (MR/CT), solid and mixed mass (BUS), mass detection (BUS), and mass size (BUS, diameter, mm) incorporated. ROMA, the Risk of Ovarian Malignancy Algorithm; BMI, body mass index; BUS, ultrasound type B; MR, magnetic resonance; CT, computed tomography.

The prognostic nomogram was formulated based on the data of multivariate regression analysis, shown in **Figure 1**. The scores of each variable of ROMA, BMI, mass boundary (MRI/CT), mass detection (BUS/MRI/CT), mass size (BUS), and mass features (BUS) were counted and summed for total points and further assessed for risks (0.1 – 0.9). For example, if there was a patient with BMI ≥26 (30 points), ROMA index was 5 (5 points), BUS detected a 4cm x 4cm mixed mass (0 + 40 + 20 points), and the MR/CT showed an unclear boundary (0 + 15 points). The total points would be 110, and the corresponding risk equaled 0.75. The higher summed points we calculated, the more malignant risk would be.

### DCA for detection of malignant ovarian tumors

**Figure 2:**
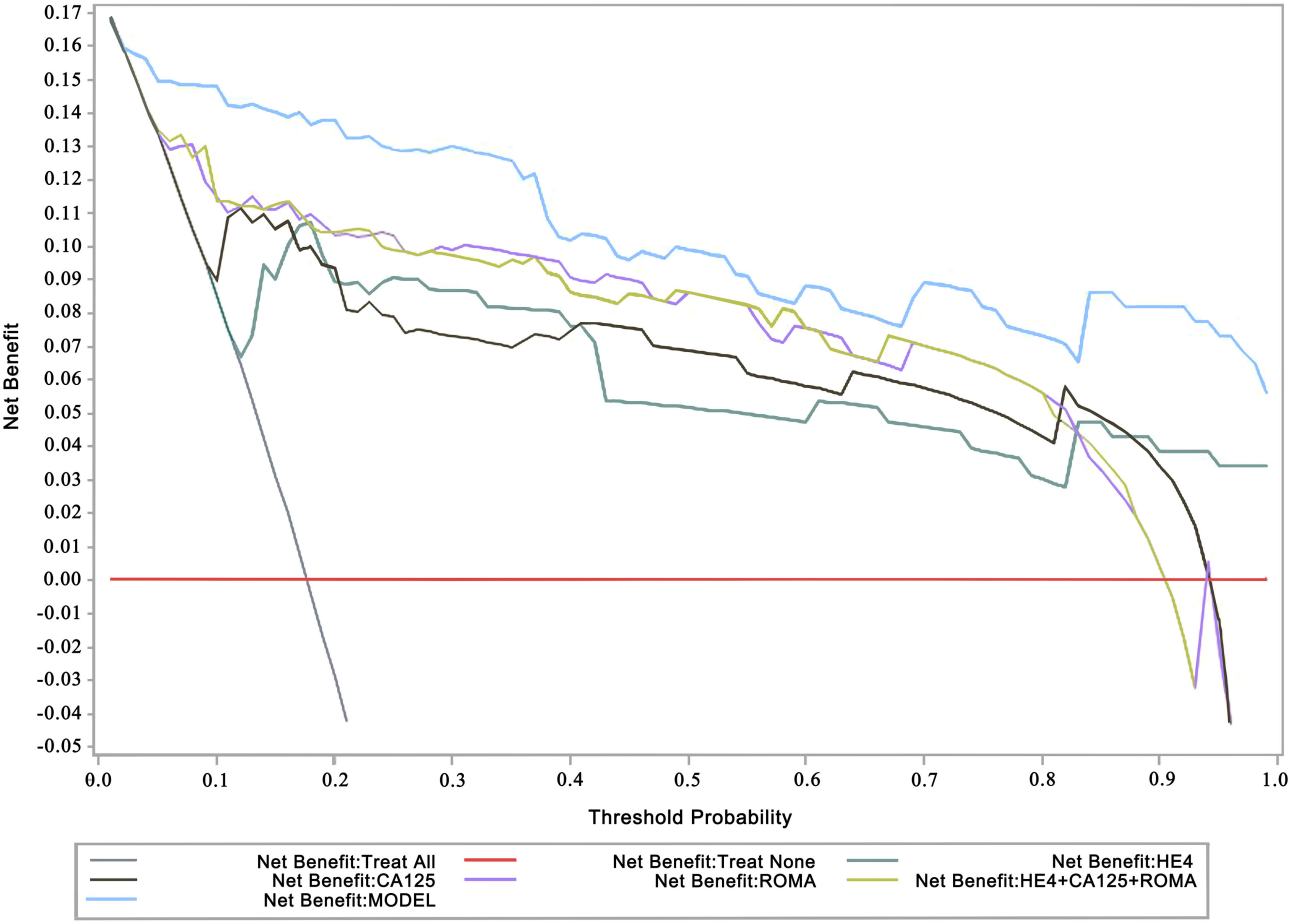
Decision curve analysis (DCA) for detection of malignant ovarian tumors. The x-axis represents the threshold probability. The y-axis measures the net benefit. The threshold probability is where the expected benefit of treatment balances the expected benefit of avoiding treatment. ROMA, the Risk of Ovarian Malignancy Algorithm; CA125, human carbohydrate antigen 125/mucin-16; HE4, human epididymis protein 4 The ROMA scores were calculated following: premenopausal women, PI=−12.0+2.38×LN [HE4]+0.0626×LN [CA125]; postmenopausal women, PI=−8.09+1.04×LN [HE4]+0.732×LN [CA125]; and ROMA (%) = exp(PI)/[1+exp(PI)]×100.

The DCA revealed that the net benefit of the calculation model was superior to the CA125, HE4, ROMA index, and HE4-CA125-ROMA index with higher threshold probabilities (**Figure 2**).

## Discussion

This study comprehensively screened out significant clinical variables associated with suspicious OT malignancy, including post-menopausal status, BMI, HE4 value, CA125 value, ROMA index, course of disease, PG-SGA, ascites, and mass locations and features, in 1,268 OT patients (*p* 0.05; **Table 1**). Then, through a multivariate logistic regression analysis, risk factors associated with malignant OT were further selected, including the ROMA index, BMI, mass boundary (MRI/CT), mass detection (BUS and MRI/CT), mass size (BUS), and mass features (BUS) (**Tables 2A-C**). Based on these variables, a prognostic nomogram prediction model with higher superiority for the detection of malignant OT was explored (**Figures 1**, **2**). To our knowledge, this was the first attempt to formulate an OC prognostic nomogram prediction model using screened significant clinical risk factors, and we believe it will be of great significance for the triage of OT patients.

Previous studies have focused on post-menopausal status, CA125 or HE4 level, ROMA index, and even modified cut-off values of serum biomarkers (7, 16–19). The diagnostic accuracy of the CA125 level, the HE4 level, or the ROMA index has varied when considering menopausal status (20). The marker CA125 is a commonly recognized oncogenic marker which is elevated in OC (21). However, it has had limited specificity due to its association with several chronic diseases such as endometriosis and coronary artery disease (CAD) (22). Furthermore, HE4 is overexpressed by OCs and has, therefore, been considered a promising biomarker for OC (23). Scaletta et al. found that serum HE4 was useful for preoperative OT diagnosis (benign vs. malignant) and also had a promising role in predicting clinical and surgical outcomes. Moreover, HE4 was better for predicting OC recurrence than CA125 alone (24). However, HE4 has been shown to be elevated in patients with renal failure and was preferably released in serous subtypes (12, 13). When combining CA125 with HE4, the ROMA index has demonstrated a higher sensitivity and specificity in OC diagnosis, especially in early-stage OC patients (15, 25–27). Therefore, the ROMA index has been approved since 2011 for the differential diagnosis and assessment of malignancy likelihood in OT women (7). Nevertheless, the findings of research related to prediction biomarkers and the ROMA index have varied worldwide due to regional and ethnic differences, and the modified cut-off values of variables have been widely explored in China, North America, and Indonesia (16, 28, 29).

Olsen et al. reported in 2013 that obesity was a risk factor for OC (30). Consistent with previous findings, the malignant rate was higher than the benign rate in our study when the BMI was ≥ 26 (*p* 0.0001). The underlying mechanism between obesity and OC might be related to hyperinsulinemia/insulin resistance and abnormalities of the insulin-like growth factor-I (IGF-I) system and signaling (31). Furthermore, malignant OTs have a shorter disease course and higher PG-SGA than that of benign OTs (*p* 0.0001, *p* = 0.0002, respectively). Unlike normal cells, it has been demonstrated that metastatic cancer cells imbalance the correlation between the ‘grow’ and ‘go’ phenotypic states and keep proliferating (32). Malnutrition might be linked with the increase of cancer-associated inflammation cytokines and the loss of muscle mass and negatively affect the prognosis of cancer patients (33).

Sayasneh et al. revealed that OC usually presented as mixed solid tissue and was frequently associated with ascites when scanned by ultrasound (34). In line with their study, we found that the rates of occurrence of ascites and solid and mixed masses detected by palpation and BUS in the malignant group of tumors were higher than that of the benign group of tumors (*p* 0.0001). Ascites in the peritoneal cavity is a hallmark of OC and contributes to patient morbidity and mortality by facilitating metastasis and contributing to chemoresistance and cell spheroid aggregation in the unique tumor microenvironment (9, 35, 36). Malignant OT often contains papillary protrusions, and later stage primary OC is usually multilocular with a high proportion of solid tissue (12, 37).

We also investigated the differences between mass distribution and mass size in malignant and benign tumors without any available pathological analysis of ovarian tissue. The rate of occurrence of a unilateral mass was higher for benign than malignant OTs, and the rate of bilateral masses was higher for malignant OTs than benign OTs. However, the size of malignant OTs was larger than that of benign OTs. Similar to our study, Riopel et al. found that benign ovarian masses were usually larger and unilateral, while malignant and metastatic OTs were more likely to be bilateral, smaller, and located in intestinal-type, mucinous ovarian masses (38, 39). Differences between our results and those of previous studies might be due to different pathological subtypes. Hence, the association between detailed OT pathological classifications and clinical manifestations will be further explored.

Except for biomarkers, several imaging strategies have been used in OT diagnosis. Ultrasonography, especially transvaginal ultrasonography (TVS), is the most commonly employed imaging modality but lacks adequate sensitivity and specificity for the early detection and assessment of adnexal masses (40, 41). CT is used to detect malignancy in an adnexal mass by exposing healthy individuals to ionizing radiation, but it demonstrates limited accuracy. Positron emission tomography with CT (PET-CT) has also been associated with physiologic uptake in normal structures, which may obscure small pelvic malignancies (42, 43). Therefore, PET/CT has not been recommended for primary cancer detection because of high false-positive rates. The MRI has shown greater accuracy and specificity in the diagnosis of malignant adnexal masses (89% and 84%, respectively) (44). However, TVS has generally been the first-line test for the conventional diagnosis of a pelvic mass due to the high cost and more limited availability of MRI (45).

Researchers have also been considering opportunities to explore a prediction model with the combination of biomarkers and imaging scans to improve OT malignancy. The risk of malignancy index (RMI), which combines TVS features, serum CA125, levels, and menopausal status, was used to characterize ovarian pathology 30 years ago (46). Recently, logistic regression models and simple rules created by the International Ovarian Tumor Analysis (IOTA) group showed a better performance than the RMI (47–49). Calster et al. assessed different neoplasms in the adnexa (ADNEX) with or without CA125 and SRRisk, considering the best models for distinguishing between benign and malignant OTs (50). However, their model included specialist test variables which made its application difficult. Funston et al. committed to building a more practical approach by incorporating tools within a 2-step pathway in which symptom-based tools were used to help select higher-risk women for specialist OC tests (51). Considering these issues, our group set up a large population to tailor a best-fit prediction module for OC based on local OT patients.

There were certain limitations to this study. First, the known OC risk factors including family history (52), hormone replacement therapy (53), ovulatory factors, such as lifetime ovulatory cycles, longer duration of breastfeeding, menstrual irregularity, and tubal ligation (54) were not involved. Therefore, a more detailed questionnaire will be generated for a future project. Second, the documented small piece could not be represented the giant population. Besides, in this study, we did not classified the sub-types of the benign or malignant masses. Therefore, it is also necessary to classify and further analyze the pathological sub-types since OC is a heterogeneous disease with variable prognoses in different sub-types (55).

However, improving on the previous research, this study enrolled participants with the majority of clinical features and screened out 7 variables to build the nomogram model for predicting OC risk with higher accuracy. The findings of our exploratory study will surely support malignant OT diagnoses and the triage of OC patients so that they may receive more timely and more precise treatment, especially during this period of the coronavirus diseazse of 2019 (COVID-19) pandemic.

## Ethics statement

The studies involving human participants were reviewed and approved by The third xiangya hospital. The patients/participants provided their written informed consent to participate in this study.

## Author contributions

BG: Data collection and assembling, data analysis and interpretation, and manuscript writing. XZ: Data analysis and interpretation, manuscript writing and revision. PG: Provision of study materials and patients. DS: Data collection and assembling. XL: Data analysis and interpretation. WL: Data collection and manuscript revision. AZ: Administrative support and manuscript revision. EP: Conception and design and administrative support. DX: Conception and design, administrative support, and manuscript revision. All authors contributed to the article and approved the submitted version.

## Funding

This study was supported by the Natural Science Foundation of Hunan Province (No. 2021JJ40956 and 2021JJ40953), the Key Research and Development Program of Hunan Province (No. 2022SK2033), the Clinical Research Center of Hunan Province (No. 2020SK4017), and the National Key Research and Development Program of China (No. 2018YFC1004800).

## Conflict of interest

The reviewer WZ declared a shared affiliation, with no collaboration, with several of the authors, XZ, PG, DS, WL, AZ, EP, DX, to the handling editor at the time of the review.

The remaining authors declare that the research was conducted in the absence of any commercial or financial relationships that could be construed as a potential conflict of interest.

## Publisher’s note

All claims expressed in this article are solely those of the authors and do not necessarily represent those of their affiliated organizations, or those of the publisher, the editors and the reviewers. Any product that may be evaluated in this article, or claim that may be made by its manufacturer, is not guaranteed or endorsed by the publisher.
